# Novel Biocompatible with Animal Cells Composite Material Based on Organosilicon Polymers and Fullerenes with Light-Induced Bacteriostatic Properties

**DOI:** 10.3390/nano11112804

**Published:** 2021-10-22

**Authors:** Sergey V. Gudkov, Alexander V. Simakin, Ruslan M. Sarimov, Alexander D. Kurilov, Denis N. Chausov

**Affiliations:** Prokhorov General Physics Institute of the Russian Academy of Sciences, Vavilova St., 38, 119991 Moscow, Russia; avsimakin@gmail.com (A.V.S.); rusa@kapella.gpi.ru (R.M.S.); ad.kurilov@gmail.com (A.D.K.); d.chausov@yandex.ru (D.N.C.)

**Keywords:** fullerene, borosiloxane, bacteriostatic agents, composite materials

## Abstract

A technology for producing a nanocomposite based on the borsiloxane polymer and chemically unmodified fullerenes has been developed. Nanocomposites containing 0.001, 0.01, and 0.1 wt% fullerene molecules have been created. It has been shown that the nanocomposite with any content of fullerene molecules did not lose the main rheological properties of borsiloxane and is capable of structural self-healing. The resulting nanomaterial is capable of generating reactive oxygen species (ROS) such as hydrogen peroxide and hydroxyl radicals in light. The rate of ROS generation increases with an increase in the concentration of fullerene molecules. In the absence of light, the nanocomposite exhibits antioxidant properties. The severity of antioxidant properties is also associated with the concentration of fullerene molecules in the polymer. It has been shown that the nanocomposite upon exposure to visible light leads to the formation of long-lived reactive protein species, and is also the reason for the appearance of such a key biomarker of oxidative stress as 8-oxoguanine in DNA. The intensity of the process increases with an increase in the concentration of fullerene molecules. In the dark, the polymer exhibits weak protective properties. It was found that under the action of light, the nanocomposite exhibits significant bacteriostatic properties, and the severity of these properties depends on the concentration of fullerene molecules. Moreover, it was found that bacterial cells adhere to the surfaces of the nanocomposite, and the nanocomposite can detach bacterial cells not only from the surfaces, but also from wetted substrates. The ability to capture bacterial cells is primarily associated with the properties of the polymer; they are weakly affected by both visible light and fullerene molecules. The nanocomposite is non-toxic to eukaryotic cells, the surface of the nanocomposite is suitable for eukaryotic cells for colonization. Due to the combination of self-healing properties, low cytotoxicity, and the presence of bacteriostatic properties, the nanocomposite can be used as a reusable dry disinfectant, as well as a material used in prosthetics.

## 1. Introduction

Nanoparticles and systems based on them are of great interest due to their promising applications in biomedical technologies. The use of metal oxide nanoparticles is one of the promising ways to overcome bacterial antibiotic resistance [1]. Fullerenes are known to have biological activity [2,3]. Antibacterial properties are also found in carbon nanomaterials, which include graphene, carbon nanotubes, fullerenes, and various forms of diamonds [4,5,6,7,8,9,10]. Moreover, it was shown that the antibacterial properties of C_60_ fullerenes are not associated with the production of reactive oxygen species (ROS), and materials based on them exert oxidative stress independent of ROS [11,12,13,14], thereby significantly reducing cytotoxicity. The ability of fullerene to photocatalyze in a nanocomposite can be used to create self-cleaning materials [15]. However, the hydrophobicity of fullerene molecules and low solubility in most organic solvents complicate their introduction into a living organism. Therefore, it can be expected that polymer materials doped with carbon nanoparticles will find application in medicine and biology as drugs, antimicrobial agents [16,17], and inhibitors (catalysts) of enzymatic processes. An important area of application of fullerene composites in a polymer matrix is the development of biological sensors [18,19,20] and bacterial biosensors [16,21,22] based on the electrically conductive and corrosive properties of fullerenes [23,24].

The antibacterial properties of fullerenes are interesting when added to a material that does not affect biomacromolecules. A biocompatible polymer matrix can act as such a material [25,26,27]. Controlled synthesis of the polymer in strictly defined nanoparticles makes it possible to control the physicochemical properties of the matrix. Borosiloxane (BS), which has adjustable stickiness [28], the ability to self-repair [29], and dissipates impact energy [30], can be a suitable polymer for creating a nanocomposite.

BS-based materials are used in various fields. Borosiloxane provides good protection of nanoparticles from physical and chemical influences and has a low production cost. Another important property of BS is the ability to quickly, like an ordinary liquid, restore integrity without any marks at the place of rupture when the separated parts are connected. This ability of BS makes it a promising material for various systems with self-healing properties. Assumptions are made about the complex multiphase structure of BS [31]. This assumption is supported by the similarity of the behavior of BS with dilatant dispersions called shear thickening fluids (STFs). Most researchers of STF materials believe that they thicken due to the formation of so-called hydroclusters [32,33,34], which are connected by hydrogen bonds. A number of researchers of BS also associate its dilatant properties with hydrogen bonds [35]. Other works prove that donor–acceptor interactions between boron and oxygen of neighboring molecules are the key reason for the manifestation of non-Newtonian properties [36].

Thus, the use of a composite based on borosiloxane and fullerene nanoparticles is of great interest for use in prostheses and biomedical devices. One of the well-known examples of the use of BS is in sports protective equipment, where materials based on it are used as shock absorbers, effectively protecting parts of the human body in such extreme sports as motorcycle and bicycle racing, alpine skiing, etc. Such materials are issued, for example, under the D3O trademark [37]. Thus, the system BS/fullerenes with antibacterial properties can be widely used in the production of sportswear.

The fields of biocompatible and self-healing electronics and display technology are relatively new trends for research and development [38,39,40,41]. In the previous works of the authors [42,43], new materials based on BS for electro-optical and electronic devices were obtained, investigated, and patented.

The aim of this work was to create a self-healing polymer material with photoinduced bacteriostatic properties, capable of adhering bacterial cells to itself. This material is based on borosiloxane and fullerene molecules. The resulting nanocomposite did not lose the basic rheological properties of borosiloxane and is capable of self-healing of the structure. When exposed to light, it is capable of generating ROS and damaging biopolymers. It shows photoinduced bacteriostatic properties and is able to adhere bacterial cells to itself. At the same time, the nanocomposite is biocompatible with mammalian cells; the surface of the nanocomposite is perfect for eukaryotic cells for colonization.

## 2. Materials and Methods

### 2.1. Fullerene C_60_ Nanoparticles Characteristics Assay

We used commercially available fullerene C_60_ nanoparticles (Sigma-Aldrich, Saint Louis, MO, USA). A hydrodynamic diameter distribution of nanoparticles was measured by dynamic light scattering with Zetasizer Ultra (Malvern Panalytical, Malvern, UK). The features of measuring the hydrodynamic radius were described earlier [44]. The spectrum of aqueous colloid of fullerene was recorded using Cintra 4040 (GBC Scientific Equipment, Braeside, Australia). The features of the optical spectrum have previously been described in detail [45].

### 2.2. Borosiloxane Composites Synthesis and Rheological Characteristics Assay

The starting materials were hydroxyl-terminated polydimethylsiloxane (PDMS) (molecular weight 20,000 g/mol) and crushed boric acid (BA) (basic substance content 99.9%, mass fraction of boric anhydride 57.1%, average particle size BA 0.075 mm). The mass ratio of PDMS and BA was 10 to 1. BS samples were obtained by heating PDMS with BA at a temperature above 200 °C. NPs were added into heating mixture of PDMS with BA. Rheological characteristics of borosiloxane and nanocomposites based on it were measured with modular compact rheometer MCR 302e (Anton Paar, Graz, Austria). To describe the non-Newtonian behavior of systems, one can apply the approach [46], which uses multiparameter rheological equations in a wide range of shear rates.

To study the effect of composite material on the properties of aqueous solutions, the composite material was heated to 40 °C and rolled through rollers. After rolling, a polymer film with a thickness of about 700–900 μm was obtained. Rectangular films with a size of 20 × 25 mm were cut from a massive workpiece. The total area of the films is approximately 10 cm^2^ (5 cm^2^ on each side). The films were placed in polypropylene vials and filled with 20 mL of water or aqueous solutions. The methodological subtleties of measuring reactive oxygen species or damage to biological macromolecules are described below.

### 2.3. Hydrogen Peroxide Concentration Measurement

The highly sensitive method of upgraded chemiluminescence in the luminol-p-iodophenol-horseradish peroxidase system was used for the quantitative evaluation of hydrogen peroxide in aqueous solutions [47]. Measures were carried out with ultrasensitive chemiluminometer Biotox-7A-USE (ANO Engineering Center—Ecology, Moscow, Russia). The samples were heated in polypropylene bottles, in a U-10 thermostat (Prüfgeräte-Werk Medigen, Berlin, Germany) at different temperatures (with an accuracy of ± 0.1 °C) for different time intervals. The concentration of the produced hydrogen peroxide was calculated using calibration curves, which were built on intensity values of chemiluminescence of samples containing known concentration of hydrogen peroxide. The initial H_2_O_2_ concentration used for calibration was evaluated spectrophotometrically with Cintra 4040 (GBC Scientific Equipment, Braeside, Australia) at a wavelength of 240 nm with a molar absorption coefficient of 43.6 (M^−1^ × cm^−1^) [48]. The samples were placed in polypropylene vials (Beckman, Bray, CA, USA) and 1 mL of a “counting solution” containing 1 cM Tris-HCl buffer pH 8.5, 50 μM *p*-iodophenol, 50 μM luminol, 10 nM horseradish peroxidase were added to determine nanomolar concentrations of H_2_O_2_. The “counting solution” was prepared immediately before the measurement. The sensitivity of the method makes it possible to determine H_2_O_2_ at a concentration of <1 nM [49].

### 2.4. OH-Radicals Concentration Measurement

OH-radicals concentration evaluation was carried out using the reaction with coumarin-3-carboxylic acid (CCA), which led to CCA hydroxylation to 7-hydroxycoumarin-3-carboxylic acid (7-OH-CCA) [50]. The 7-OH-CCA is a convenient fluorescent probe for determining the formation of these radicals. A 0.2 M phosphate buffer (pH 6.8) was added to a CCA solution in water (0.5 mM, pH 3.6). Experimental samples (and control) were heated in polypropylene bottles in a U10 thermostat (Berlin, Germany) at a temperature of 80 ± 0.1 °C for 2 h. The fluorescence of 7-OH-CCA (the product of the reaction of CCA with a hydroxyl radical) was measured on a JASCO 8300 spectrofluorimeter (JASCO, Tokyo, Japan) with λ_ex_ = 400 nm, λ_em_ = 450 nm. Calibration was performed using commercial 7-OH-KKK [51].

### 2.5. Long-Lived Reactive Protein Species Concentration Measurement

A chemiluminescence method is an effective and sensitive technique for determining free radical reactions [52]. In this case the interaction of radicals, energy is emitted in the form of light quanta. The study of long-lived reactive protein species was carried out by measuring the chemiluminescence of protein solutions induced by an increase in temperature using a Biotox-7A chemiluminometer (ANO Engineering Center—Ecology, Russia). The measurements were performed in the dark, at room temperature, in 20 mL plastic polypropylene vials for liquid scintillation counting (Beckman, Bray, CA, USA). The use of large volumes in comparison with standard (0.1 mL) volumes increased the method sensitivity by almost 200 times [53]. All samples had been kept in the dark at room temperature for 30 min after exposure to X-ray radiation. The proteins which were not exposed to heat served as controls. The method was described in more detail earlier [54].

### 2.6. Enzyme-Linked Immunosorbent Assay (ELISA)

A non-competitive enzyme-linked immunosorbent assay (ELISA) with using monoclonal antibodies specific to 8-oxoguanin (anti-8-OG antibodies) was developed for the quantitative measurement of 8-oxoguanine in DNA [55]. DNA samples (350 μg/mL) were denatured by boiling in a water bath for 5 min and cooled in ice for 3–4 min. Aliquots (42 μL) were applied to the bottom of the wells of the ELISA plates. DNA was immobilized using a simple adsorption procedure with incubation for 3 h at 80 °C until the solution was completely dry. Nonspecific adsorption sites were blocked by 300 μL of a solution containing 1% skimmed milk powder in 0.15 M Tris-HCl buffer, pH 8.7, and 0.15 M NaCl. Further the plates were incubated at room temperature overnight (14–18 h). The antigen-antibody complex formation with anti-8-OG antibodies (at a dilution of 1:2000) was carried out in a blocking solution (100 μL/well) by incubation for 3 h at 37 °C. Washed twice (300 μL/well) with 50 mM Tris-HCl buffer (pH 8.7) and 0.15 M NaCl with 0.1% Triton X-100 after 20 min incubation. Further a complex with conjugate (anti-mouse immunoglobulin labeled with horseradish peroxidase (1:1000)) was formed by incubating for 1.5 h at 37 °C in a blocking solution (80 μL/well). Then the wells were washed 3 times as described above. Further a chromogenic substrate containing 18.2 mM ABTS and hydrogen peroxide (2.6 mM) in 75 mM citrate buffer, pH 4.2 (100 μL/well) were added in each well. The reactions were stopped by adding an equal volume of 1.5 mM NaN_3_ in 0.1 M citrate buffer (pH 4.3) upon reaching color. The optical samples density was measured on a plate photometer (Titertek Multiscan, Vantaa, Finland) at λ = 405 nm. The method is described in more detail earlier [56].

### 2.7. Bactericidal Activity Assay

Experiments in a cultural environment. Gram-negative bacteria *Escherichia coli* were cultured. Using aseptic techniques, we carefully transferred a 5 mL aliquot of LB broth into a sterile, lidded glass culture tube [57]. Using a sterile applicator stick, one well-isolated colony was transferred from the solid medium plate to the culture tube. Then the colony was resuspended in a glass culture tube. To determine the concentration of bacteria, a spectrophotometric study was carried out. The optical density of the resulting medium was determined using a drop spectrophotometer UV5Nano Excellence (Mettler Toledo). For analysis, 10 μL of the medium containing the bacteria was irreversibly taken. After determining the concentration of bacteria, the resulting concentrated medium containing bacteria was diluted in a larger volume. For the experiments, films of a composite material with a thickness of 700–900 μm and a size of 10–15 mm were made. The film was sterilized by soaking three times in ethyl alcohol for 30 min. After that, the film was put on a round sterile hoop. A nutrient medium with bacteria was poured into the hoop, and the top of the hoop was sealed with a piece of glass slide. The resulting structure was placed in an ES-20 incubator shaker (Biosan) (37 °C, approximately 150 rpm). During incubation, the concentration of bacteria was estimated using microscopy and an algorithm developed by us for determining optically dense objects in the frame. At the end of the experiment, the structures were disassembled and the concentration of bacteria was estimated again using a drop spectrometer. 

Experiments on the surface of a solid substrate. Initially, LB agar was prepared by weighing the appropriate powder medium, agar and water into a sterile flask. The medium was autoclaved. In a laminar, approximately 25–30 mL of LB agar was poured into sterile Petri dishes. Thereafter, the agar plates were allowed to solidify. Sometimes agar plates were stored upside down at 4 °C for several days. During the experiment, about 100 μL of a suspended culture of E. coli was added to a Petri dish with LB agar. The culture was spread over the entire dish using a sterile glass spatula. The Petri dish is incubated for several hours at 37 °C. After that, using a heated composite material, we tried to transfer bacterial cells from the surface of the substrate to the surface of the material. The concentration of microorganisms remaining on the surface was determined using microscopy. Microorganisms stained using a crystal violet indicator and examined under a microscope at 1000 magnification. The details of the experiment were described earlier [58]. Since we used a drop spectrophotometer (optical path length of about 50 μm) and microscopy, we reported the concentration of microorganisms in the number of bacteria per unit area.

### 2.8. Cell Culture

Biocompatibility studies were performed using standard in vitro test systems. Human neuroblastoma SH-SY5Y cell culture was used as a standard cell model. Initially, the SH-SY5Y cell line was subcloned from the SK-N-SH cell line isolated from the bone marrow material of a four-year-old female patient with neuroblastoma. SH-SY5Y cells are a classic model of neuronal function and in vitro differentiation. SH-SY5Y cells usually grow in tissue culture in two different ways, which is why they are used in the study of cell differentiation. The differentiation is the process of implementing a genetically determined program for the formation of a specialized cell phenotype, reflecting their ability to certain profile functions. SH-SY5Y cells are also interesting in that they can grow not only in monolayers, but also form cell aggregates, which also take root on substrates. Moreover, SH-SY5Y cells in vitro can spontaneously inter-contain two phenotypes, neuroblast-like cells and epithelial-like cells [59].

**Figure 1:**
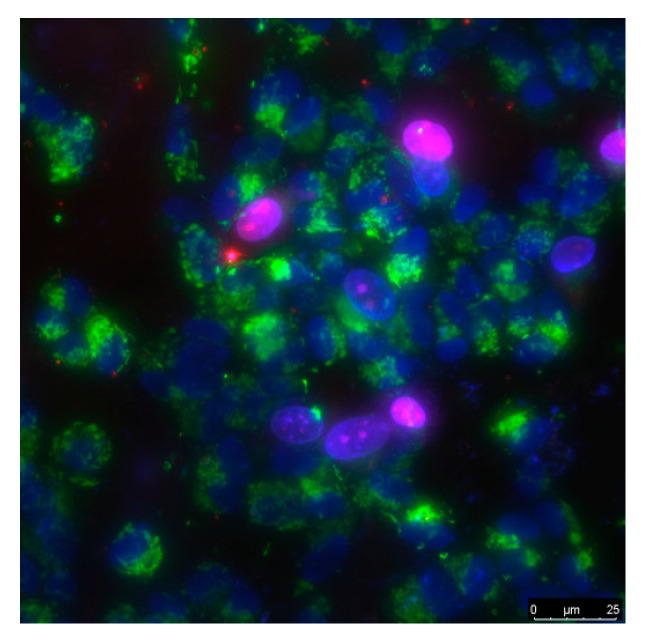
The sample of cell culture micrograph. The mitochondria of the cells are colored green; they can be used to estimate the size of the cells. Normal cell nuclei are colored blue. Nuclei of non-viable cells are stained in purple.

The cells were grown in DMEM medium (Biolot, Ankara, Turkey) supplemented with 10% fetal calf serum (Gibco, Waltham, MA, USA), 30 μg/mL gentamicin, at 37 °C and 5% carbon dioxide in a CO_2_ incubator (Binder, Germany). Fragments of material samples 20 × 20 mm in size were placed in Petri dishes with diameter 35 mm, 1 sample pre dish. Then, on the surface of material samples, cells were inoculated at a concentration of 10^4^ cells/cm^2^, in a volume of 3 mL per dish. Cells were cultured on samples during 3 days. Cells growing on the samples surface were stained with fluorescent dyes 2 μg/mL Hoechst 33342 (Sigma, Saint Louis, MO, USA) and 2 μg/mL propidium iodide (Sigma, USA) to determine the numbers of living and dead cells respectively. Hoechst 33342 stains all cells (live and dead). The propidium iodide dye penetrates into living cells extremely slowly; therefore, during the short incubation time (we used about 10 min) it stains only cells with a damaged plasma membrane. The plasma membrane with breaks leading to dye penetration was one of the main criteria for determining that the cell was dead. Thus, Hoechst 33342 stains both living and dead cells, while propidium iodide only stains dead cells (Figure 1). Microscopic assay of the samples was carried out with imaging system based on Leica DMI6000 (Leica, Berlin, Germany). On the surface of each sample at least 500 cells were counted for analysis [60].

The mitotic index of cells in the logarithmic growth phase (3 days from the moment of seeding) was used to analyze proliferation of cells. The number of cells in a state of mitosis was determined using fluorescence microscopy using in vitro staining with the Hoechst 33342 fluorescent dye (Sigma, USA). Mitotic cells were identified by the chromatin distribution characteristic of prophase (P), metaphase (M), anaphase (A), and telophase (T). For analysis, at least 500 cells were counted on each sample surface. The mitotic index (MI) was calculated by the formula MI = (P + M + A + T)/N × 100%, where (P + M + A + T) is the number of cells at the stage of prophase, metaphase, anaphase, and telophase, respectively, and N is the total number of analyzed cells [61].

### 2.9. Exposure to Visible Light

**Figure 2:**
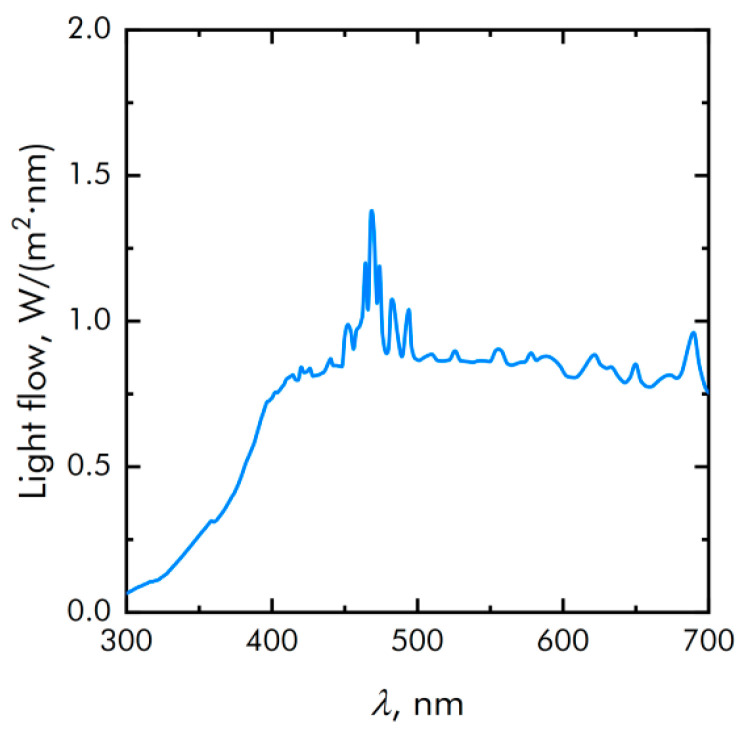
The spectrum of the light source.

A laboratory illumination system based on a 150-W XBO150W/4 xenon lamp (OSRAM, Munich, Germany) was used as a light source. The spectral characteristics of the setup in various operating modes (including the modes when films were tested) were recorded using a PM200 radiation power/energy meter complex (Thorlabs, Newton, NJ, USA). The spectrum of the light source is shown in Figure 2.

### 2.10. Statistic

The data were analyzed using GraphPad Prism eight and Origin software and were presented as means ± SEM. Data from at least three independent experiments were used for averaging.

## 3. Results

### 3.1. Physicochemical Characteristics of Materials and Composite

**Figure 3:**
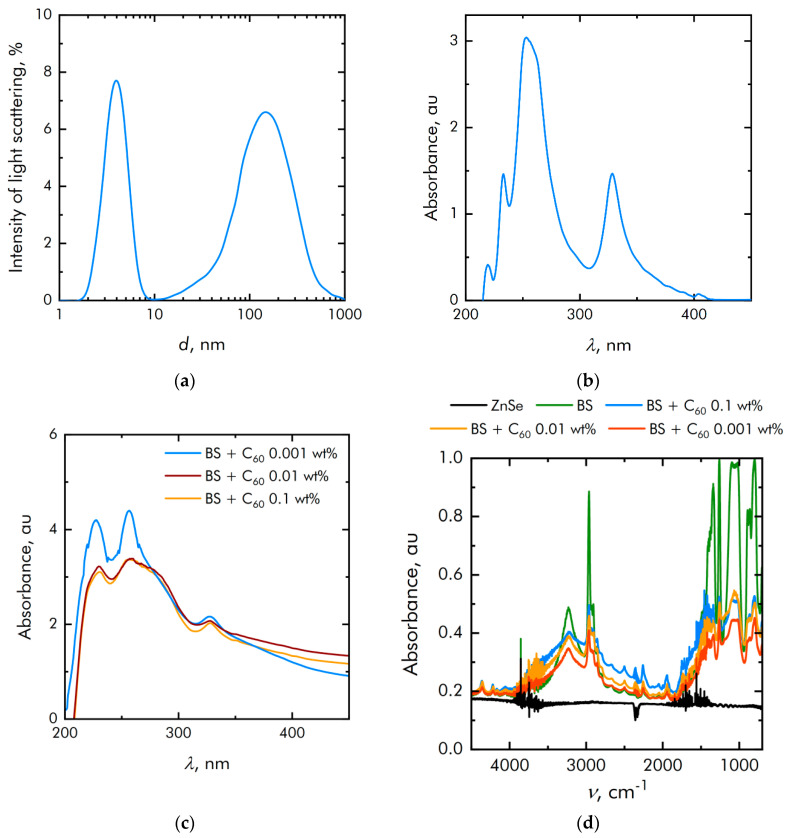
Physicochemical properties of fullerenes: (**a**) Particle size distribution of fullerenes; (**b**) Optical absorption of a colloidal solution of fullerenes; (**c**) Optical absorption of a colloidal solution of BS + fullerenes; (**d**) Characterization of the obtained polymers by FTIR methods.

Dynamic light scattering studies have shown that the particle size distribution is unimodal and rather narrow (Figure 3a). The average hydrodynamic particle diameter is about 1.4 μm. Figure 3b shows the absorption spectrum of a colloidal solution of heptane and fullerenes with a control sample of pure heptane. It is shown that the absorption spectrum corresponds to the spectrum of fullerenes. The absorption spectrum of BS + C_60_ solution in heptane with same control sample is shown in Figure 3c. It is clearly seen that the spectral absorption maxima do not shift when fullerenes are added to the polymer matrix. The absorption spectrum of the composites was also determined using the FTIR (Figure 3d). The polymer was deposited in a thin layer on a ZnSe plate, which is why the spectra show combs of narrow spikes characteristic of ZnSe (for example, in the range 4000–3500 cm^−1^). In general, the spectra differ from each other in proportion to the fullerene concentration in the composite material.

**Figure 4:**
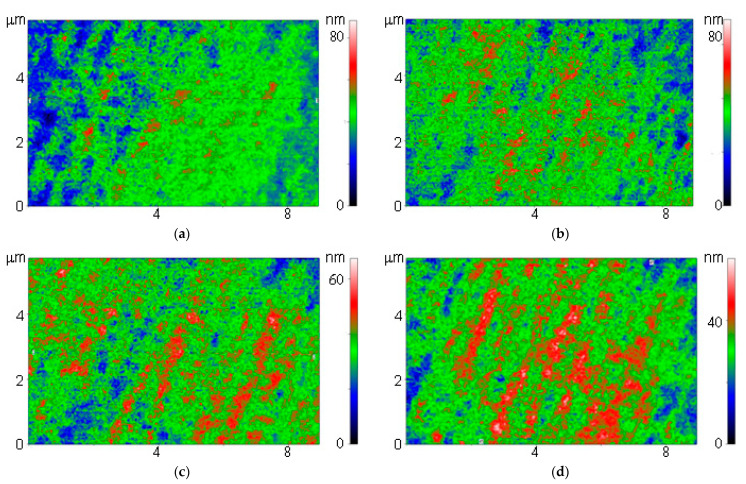
Images obtained on a polymer without fullerenes (**a**) and polymers with fullerenes at a concentration of 0.001% (**b**), 0.01% (**c**) and 0.1% (**d**) using a modulation interference microscope.

Borsiloxane and fullerenes differ significantly in their optical properties. In this regard, we used a modulation interference microscope. This microscope allows you to create two-dimensional maps that show how the phase incursion of the wave changes. In the case of optically opaque objects, the microscope works as a high-precision profilometer. Using modulation interference microscopy, it was shown that on borosiloxane and composites based on borosiloxane and fullerenes, areas in the form of oriented bands are observed (Figure 4). Moreover, the intensity of the bands increases with an increase in the concentration of fullerenes in the composite.

We also tried to characterize the polymer using SEM. Nanocomposite does not have sufficient electrical conductivity; therefore, it was not possible to use the SEM method without metal deposition on it. When gold was sprayed onto the surface, the surface began to change. The polymer is probably too soft and too fusible. We also tried to characterize the composite using AFM. It turned out that available cantilevers sink and get stuck, both in pure polymer and in composites based on it. 

**Figure 5:**
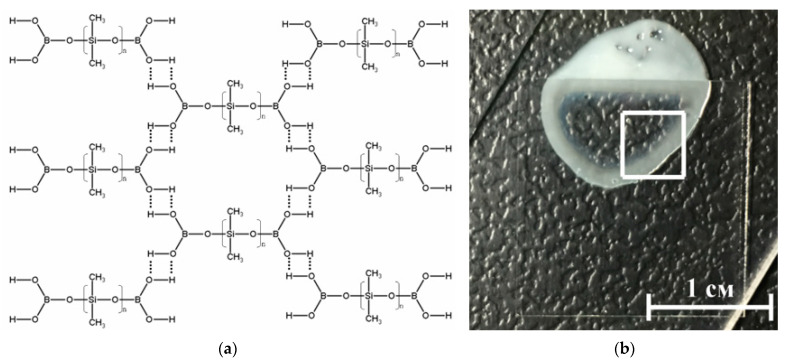
(**a**) Schematic representation of the structure of the BS. BS molecules linked through hydrogen bonds; (**b**) Photo BS-VT, where part of the sample is under the cover glass.

A borosiloxane (BS) based material was selected as the particle matrix (Figure 5). The use of borosiloxane as a polymer matrix is due to the presence of “smart” properties (self-healing, variable elasticity) and the possibility of simple adjustment of mechanical properties at the synthesis stage. Borosiloxane can be in both solid and liquid aggregate state, as well as have a viscous, rubber-like or glassy consistency depending on the synthesis conditions. From the point of view of the molecular structure, B_512_Ss belong to the class of organosilicon compounds containing the R-Si-O-B group, where R is a hydrocarbon radical. Depending on the molecular structure and molecular weight of the starting organosiloxane, the conditions for the synthesis of BS, as well as the amount and properties of the introduced functional additives (fillers, plasticizers, thickeners, etc.), BS-based materials can have different mechanical properties. They can be in both solid and liquid state of aggregation, and also have a viscous (Figure 3), rubbery or glassy consistency [62]. Highly elastic behavior is realized due to stretching of siloxane fragments, and viscous due to the fact that these formed layers can freely move relative to each other. Thus, brittle destruction of BS and composites based on it can occur many times without changing the characteristics of the material and the molecular weight of its constituent molecules, since there is no destruction of covalent bonds.

**Figure 6:**
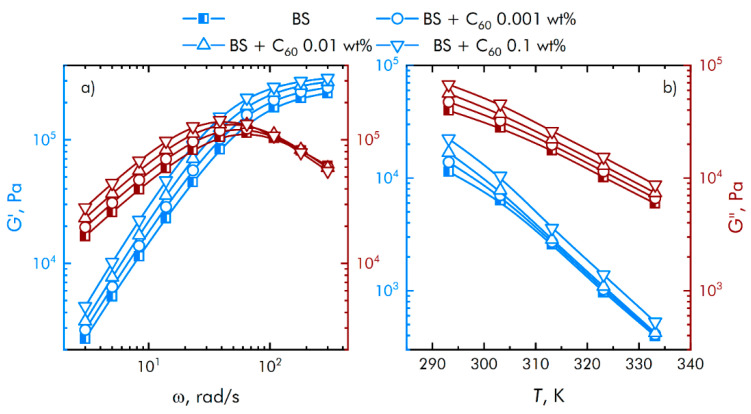
Influence of composite material based on boroxiloxane and fullerenes on the mechanical spectra; storage modulus—blue symbol, loss modulus—red symbol. Influence of the angular velocity of movement of the measuring surfaces on the mechanical properties of nanocomposites (**a**). Effect of temperature on the mechanical properties of nanocomposites (**b**).

Assessment of the rheological characteristics of BS and systems based on it is important for the possibility of their further application in bioactive substances. Figure 6 shows the concentration dependences of the real and imaginary parts of the dynamic modulus of elasticity at different concentrations of fullerene particles. With an increase in the concentration of fullerenes, an increase in the viscoelastic properties is observed. BS has complex rheological responses when shear loads are applied due to microstructural reorganization.

From Figure 5 it can be concluded that at low shear rates the prevalence of the viscous properties of the BS over the elastic ones is observed, with an increase in the speed, the contribution of the viscous properties decreases, while the storage modulus reaches a constant value. In this case, the region of transition from viscous to elastic properties is adjusted at the stage of borosiloxane synthesis, depending on the amount and properties of the introduced functional additives.

### 3.2. Effect of Composite on ROS Generation and Damage to Biopolymers

**Figure 7:**
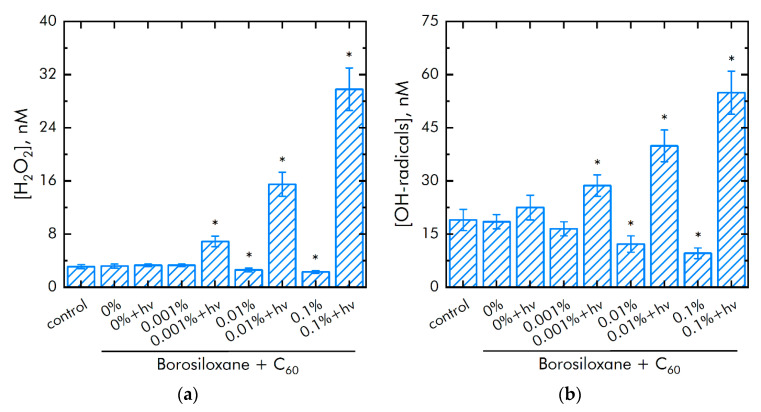
Effect of composite material containing borosiloxane and fullerenes on the generation of reactive oxygen species: (**a**) Formation of hydrogen peroxide (2 h, 40 °C); (**b**) Generation of hydroxyl radicals (2 h, 80 °C); * indicate a significant difference at 5% level in comparison with the control (*p* < 0.05). Data are presented as mean values and standard errors.

The effect of polymer and fullerene particles on the generation of hydrogen peroxide, as the most stable representative ROS (Figure 7a), and hydroxyl radicals, as the most reactive ROS (Figure 7b), was studied. It was shown that borosiloxane has no effect on the generation of hydrogen peroxide. To estimate a light influence on fullerene bacteriostatic and cytotoxic actions we use an experimental protocol with dark and light samples (indicated as “fullerenes + *hv*”). A composite material based on borosiloxane and fullerenes + *hv* increases the rate of hydrogen peroxide generation by 2 times at a particle mass concentration of 0.001%.

The rate of generation of hydrogen peroxide increases by 9 times with an increase in the mass concentration of particles 0.1%. Composite borosiloxane with fullerenes (in dark) decreased hydrogen peroxide production by 20 and 30% at particle concentrations of 0.01 and 0.1%, respectively. Borosiloxane has no effect on the generation of hydroxyl radicals. In this case, a composite material based on borosiloxane and fullerenes + *hv* increases the rate of generation of hydroxyl radicals by 28, 77 and 250% at a particle concentration of 0.001, 0.01, and 0.1%, respectively. Composite borosiloxane with fullerenes (in dark) decreased hydrogen peroxide production by 40 and 60% at particles concentration 0.01 and 0.1%, respectively.

**Figure 8:**
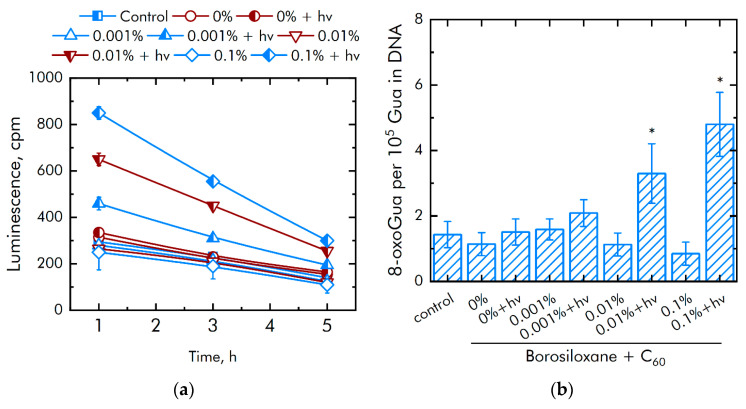
Effect of composite material containing borosiloxane and fullerenes on the generation on biomacromolecules damage: (**a**) Formation of long-lived reactive protein species (2 h, 40 °C); (**b**) Generation of 8-oxoguanosine (2 h, 45 °C); * indicate a significant difference at 5% level in comparison with the control (*p* < 0.05). Data are presented as mean values and standard errors.

It is known that excessive ROS generation is associated with the damage to biomacromolecules. The effect of a composite material based on borosiloxane and fullerenes on the formation of long-lived active forms of proteins (LRPS) was investigated (Figure 8a). The borosiloxane without fullerenes did not affect the generation or decay rate of long-lived active forms of proteins. All variants of nanocomposites in the dark did not influence long-lived active forms of protein production. The borosiloxane with fullerenes + *hv* increased significantly the rate of formation of long-lived active forms of proteins. An increase in the rate by 37, 91, and 250% is observed at a fullerene concentration of 0.001, 0.01 and 0.1%, respectively. The fullerenes have almost no effect on the average half-life of active forms of proteins. In all groups, the half-life is about 4–5 h.

The effect of a composite material based on borosiloxane and fullerenes on the formation of 8-oxoguanine in DNA in vitro was investigated (Figure 8b). It was found that borosiloxane did not affect the formation of 8-oxoguanine in DNA in vitro. It was found that at a fullerene concentration of + *hv* 0.001, 0.01, and 0.1% increased the rate of 8-oxoguanine production in DNA by 38, 200 and 300%, respectively. All variants of nanocomposites in the dark did not influence 8-oxoguanine production in DNA.

### 3.3. Bacteriostatic Properties of the Composite

**Figure 9:**
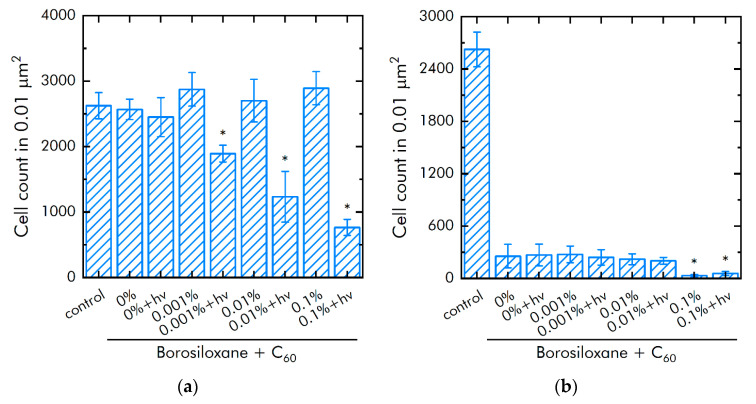
Influence of composite material based on borosiloxane and fullerenes on the growth and development of *E. coli*: (**a**) Development of *Escherichia coli*. Incubation time is 24 h; (**b**) Effect of tearing off bacteria from a substrate using a composite material based on borosiloxane and fullerenes; * indicate a significant difference at 5% level in comparison with the control (*p* < 0.05). Data are presented as mean values and standard errors.

The effect of fullerenes in borosiloxane on the growth of *E. coli* was studied (Figure 9a). The borosiloxane without fullerenes did not affect the growth and growth of *E. coli* bacteria. It was found that when fullerenes + *hv* appear in the polymer, the density of bacterial cultures grown on the composite decreases by 23, 50, and 69% at a particle concentration of 0.001, 0.01, and 0.1% at a particle concentration respectively. All variants of nanocomposites in dark did not influence on *E. coli* growth.

In a separate series of experiments, the bacteriostatic properties of an aqueous colloidal solution of C_60_ were investigated. The concentrations studied were 0.001, 0.01, and 0.1%. It was shown that at all studied concentrations in the dark, fullerenes did not affect the growth and development of bacteria. When exposed to light, fullerenes significantly affected the growth and branching of bacteria. Thus, at a concentration of 0.001 and 0.01% fullerenes in the medium and constant illumination, the growth of the number of bacteria in the medium was not observed. At a minimum concentration of 0.0001%, the density of the bacterial culture was 76% less compared to the illuminated control.

The effect of a composite material based on borosiloxane and fullerenes on the detachment of *E. coli* bacteria from the substrate was studied (Figure 9b). The borosiloxane without fullerenes effectively detached the *E. coli* bacteria from the substrate. The number of bacteria on the substrate decreases by 10 times. The addition of fullerenes + *hv* to the polymer in mass concentrations of 0.001 and 0.01% has no significant effect. With an increase in the concentration of fullerenes in the polymer to 0.1%, the detachment of bacteria from the substrate occurs 5 times more efficiently as compared to pure borosiloxane. The number of bacteria on the substrate is reduced by 45 times. There were no differences between bacterial detachment in dark and light.

### 3.4. Biocompatibility with Mammalian Cells

**Figure 10:**
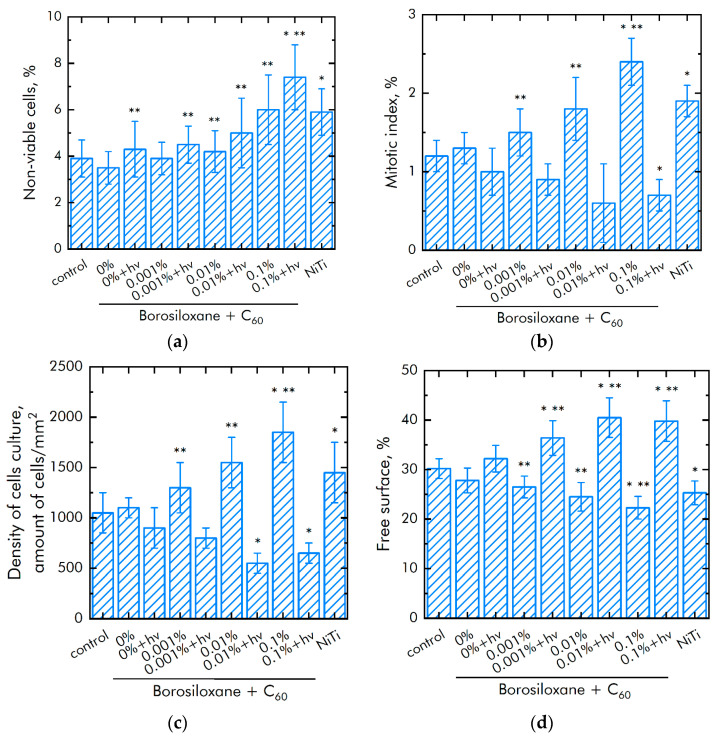
Effect of composite material based on borosiloxane and fullerenes on the main characteristics of cell culture growth and development: (**a**) Cell viability; (**b**) Mitotic index of a cell population; (**c**) Cell culture density; (**d**) Rate of spreading; * indicates a significant difference at 5% level in comparison with the control (*p* <0.05). ** indicates a significant difference at 5% level in comparison with the NiTi group (*p* < 0.05). Data are presented as mean values and standard errors.

The effect of a composite material based on borosiloxane and fullerenes on the viability of mammalian cells was investigated (Figure 10a). The number of non-viable cells grown on control substrates and culture plastic did not exceed 4%. Approximately the same number of non-viable cells was observed on a composite material based on borosiloxane without fullerenes or contains 0.001% of fullerenes particles. At the same time, when nitinol medical alloy is used as a substrate, an almost 50% greater number of nonviable cells is observed (approximately 6%). Number of nonviable cells were 5 and 7.5% on a composite material based on borosiloxane and fullerenes + *hv* in concentration 0.01 and 0.1%, respectively.

The mitotic index of cells in the logarithmic growth phase was estimated to analyze the ability of cells to divide (Figure 10b). The mitotic index of cells growing on the titanium sample surface was 1.2%. The mitotic index on nitinol was 1.9%. The mitotic index was 0.6–1% in case of composite material based on borosiloxane and fullerenes + *hv*. The mitotic index was 1.5–2.5% in case of composite material based on borosiloxane and fullerenes in the dark.

The cell culture density averages 1050 cells per mm^2^ on titanium (Figure 10c). The density reaches 1450 cells per mm^2^ on a nitinol substrate. The cell density reaches 550–900 cells per mm^2^ on a composite material based on borosiloxane and fullerenes + *hv* and 1300–1800 cells per mm^2^ on the composite in dark. After 72 h of culturing cells on the surface of the materials, a morphological analysis was carried out. It was found that the surfaces of the composite material based on borosiloxane and fullerenes are more suitable for cell attachment and spreading (Figure 10d). Moreover, the degree of suitability is comparable to culture plastic and the medical alloy nitinol. At the same time, after 72 h of cultivation on the surface of all samples of materials, the cells do not form a merged monolayer. Only some of its elements are observed. On the surface of all materials, cells occupy about 70–75% of the surface available for growth.

Table 1 presents data on the effect of a colloidal solution of fullerene nanoparticles on the growth and development of eukaryotic cells. In these experiments, the cells were in contact with fullerenes, but not in contact with the polymer. Fullerenes were in the culture medium, and not on the surface of the polymer. It has been shown that fullerenes affect the number of viable cells and the value of the mitotic index at significantly lower concentrations as compared to the polymer containing fullerenes. This is probably due to the fact that when fullerenes are found in deep layers of the polymer, most of the C_60_ molecules do not affect mammalian cells.

## 4. Discussion

The ROS generation in many cases can be considered as a negative effect and a key factor of toxicity when using metal oxide particles in biomedicine [63,64]. Nevertheless, the possibility of generation and quenching of ROS by fullerenes has been reported [65,66,67,68,69,70]. Our results show that the use of borosiloxane as a polymer matrix does not affect the generation of ROS. At the same time, when composite samples are irradiated with light, a significant acceleration of ROS formation is observed, in particular, at a concentration of 0.1 wt%, the rate of generation of hydrogen peroxide increases by 9 times, and the rate of generation of hydroxyl radicals by 2.5 times. Thus, light-induced acceleration of ROS formation was detected in the studied composite. This ability is crucial in biological applications of the developed material, as it allows adjustment of the balance between toxicity and bacteriostatic properties of the composite.

A reliable marker of oxidative damage to biomacromolecules by ROS is the level of long-lived active forms of proteins and 8-oxoguanine in DNA [71,72,73,74,75,76]. Our study showed that borosiloxane does not affect the level of long-lived active forms of proteins and 8-oxoguanine in DNA in vitro. The presence of fullerenes in the polymer matrix slightly reduces the level of LRPS and 8-OH-Gua. Irradiation with light similarly to ROS causes the opposite effect: at concentration of 0.1 wt% fullerenes, the level of LRPS and 8-OH-Gua increases 2.5 and 3 times, respectively, in comparison with pure borosiloxane. The toxicity level data obtained in our study is 2–3 times lower than in the works of other authors [77].

It is known that fullerenes have high bacteriostatic properties not only against *E. coli* bacteria, but also against bacteria *B. subtilis*, *S. Aureus*, *S. epidermidis*, *E. hirae*, *S. typhi*, *P. acnes*, and *E. faecalis* [11,12,13,78,79,80,81,82,83,84,85]. The use of borosiloxane as a carrier increases the detachment of bacteria from the substrate by one order of magnitude, and the incorporation of 0.1 wt% fullerenes decreases the density of bacterial structures by 3 times (under light irradiation) and increases the detachment of bacteria five-fold. The bacteriostatic efficacy of the composites obtained by us turned out to be comparable with the results obtained by other authors.

In the present study, we researched the effect of the developed composite on both the viability of mammalian cells and the ability to divide according to the mitotic index of cells in the logarithmic growth phase. Borosiloxane matrix has no significant effect on cell viability and division. The addition of 0.001–0.01% fullerenes did not have a cytotoxic effect on cells or cell feeding in the dark, which is consistent with the literature data on the low cytotoxicity and hemolytic activity of fullerenes [86,87]. Irradiation of 0.1 wt% fullerenes leads to an increase in non-viable cells by 70% and a decrease in the mitotic index by 30%. In this case, the mitotic index of the non-irradiated sample is 3.5 times higher than that of the irradiated one.

Thus, the light-induced bacteriostatic activity of the composite based on borosiloxane and fullerenes was found. At the same time, the used polymer matrix does not have a cytotoxic effect and is biocompatible. The mechanical properties of a composite are mainly determined by the polymer matrix and practically do not depend on the mass content of particles, and strict control of the viscoelastic properties of composites for various tasks is carried out even at the stage of polymer synthesis.

The developed material, in contrast to composites based on metal oxide nanoparticles, does not cause a constant increased content of ROS, and makes it possible to control the level of cytotoxicity in a non-contact way (visible light). The results obtained in this study will aid in the design of more efficient targeted biocides in the future.

## 5. Conclusions

In the present study, a composite based on borosiloxane and fullerenes for biomedical applications was synthesized and characterized. Borosiloxane provides good protection against physical and chemical damage to particles and has a low production cost. The resulting material exhibits strong light-induced bacteriostatic properties by the example of *E. coli* culture and has low cytotoxicity. In this case, the polymer matrix does not affect either the growth and the development of bacteria or the viability of mammalian cells. The mechanical properties of the composite at such low concentrations of the dopant are almost completely determined by the properties of the polymer and can be tuned at the stage of synthesis. The use of borosiloxane as a carrier increases the detachment of bacteria from the substrate by one order of magnitude, and the incorporation of 0.1 wt% fullerenes decreases the density of bacterial structures trebled (under light irradiation) and increases the detachment of bacteria five-fold. Thus, a synergistic effect is observed, which makes it possible to reduce the amount of incorporated fullerenes (and, accordingly, toxicity) while maintaining the high bacteriostatic properties of the composite. The resulting composite, based on borosiloxane and fullerenes, is of great interest for use in prostheses and biomedical devices. A significant increase in bacterial detachment, together with bacteriostatic properties, makes the developed material especially attractive for the use as a reusable dry disinfectant.

## Figures and Tables

**Table 1:** The effect of a colloidal solution of C_60_ on the growth and development of eukaryotic cells.

Concentration of C_60_, %	Cell Culture Parameter	
	Viable Cells, %	Mitotic Index, %
Control	95.9 ± 1.5	1.2 ± 0.4
Control + *hv*	94.3 ± 1.9	1.3 ± 0.2
0.0001%	95.6 ± 2.1	1.4 ± 0.3
0.0001% + *hv*	95.3 ± 2.3	1.1 ± 0.4
0.001%	95.7 ± 1.7	1.5 ± 0.3
0.001% + *hv*	93.2 ± 1.8	0.9 ± 0.4
0.01%	94.2 ± 2.5	1.9 ± 0.2 *
0.01% + *hv*	92.1 ± 2.1 *	0.7 ± 0.2 *

*—indicates a significant difference at 5% level in comparison with the control (*p* < 0.05).

## Data Availability

The raw data supporting the conclusions of this article will be made available by the authors, without undue reservation.

## References

[1] Gudkov S.V., Burmistrov D.E., Serov D.A., Rebezov M.B., Semenova A.A., Lisitsyn A.B. (2021). Do iron oxide nanoparticles have significant antibacterial properties?. Antibiotics.

[2] Ye L., Kollie L., Liu X., Guo W., Ying X., Zhu J., Yang S., Yu M. (2021). Antitumor Activity and Potential Mechanism of Novel Fullerene Derivative Nanoparticles. Molecules.

[3] Gataullin A.R., Bogdanova S.A., Galyametdinov Y.G. (2019). Dispersion of fullerene C_60_ in organized media. Liq. Cryst. Appl..

[4] Maas M. (2016). Carbon nanomaterials as antibacterial colloids. Materials.

[5] Wang X., Liu X., Han H. (2013). Evaluation of antibacterial effects of carbon nanomaterials against copper-resistant *Ralstonia solanacearum*. Colloids Surf. B Biointerfaces.

[6] Elias L., Taengua R., Frígols B., Selesa B., Serrano-Aroca A. (2019). Carbon nanomaterials and LED irradiation as antibacterial strategies against gram-positive multidrug-resistant pathogens. Int. J. Mol. Sci..

[7] Xin Q., Shah H., Nawaz A., Xie W., Akram M.Z., Batool A., Tian L., Jan S.U., Boddula R., Guo B. (2019). Antibacterial carbon-based nanomaterials. Adv. Mater..

[8] Hu W., Peng C., Luo W., Lv M., Li X., Li D., Huang Q., Fan C. (2010). Graphene-based antibacterial paper. ACS Nano.

[9] Azizi-Lalabadi M., Hashemi H., Feng J., Jafari S.M. (2020). Carbon nanomaterials against pathogens; the antimicrobial activity of carbon nanotubes, graphene/graphene oxide, fullerenes, and their nanocomposites. Colloid Interface Sci..

[10] Yousefi M., Dadashpour M., Hejazi M., Hasanzadeh M., Behnam B., de la Guardia M., Shadjou N., Mokhtarzadeh A. (2017). Anti-bacterial activity of graphene oxide as a new weapon nanomaterial to combat multidrug-resistance bacteria. Mater. Sci. Eng. C.

[11] Lyon D.Y., Brunet L., Hinkal G.W., Wiesner M.R., Alvarez P.J. (2008). Antibacterial activity of fullerene water suspensions (nC_60_) is not due to ROS-mediated damage. Nano Lett..

[12] Lyon D.Y., Adams L.K., Falkner J.C., Alvarez P.J. (2006). Antibacterial activity of fullerene water suspensions: Effects of preparation method and particle size. Environ. Sci. Technol..

[13] Lyon D.Y., Alvarez P.J. (2008). Fullerene water suspension (nC_60_) exerts antibacterial effects via ROS-independent protein oxidation. Environ. Sci. Technol..

[14] Reynoso E., Durantini A.M., Solis C.A., Macor L.P., Otero L.A., Gervaldo M.A., Durantini E.N., Heredia D.A. (2021). Photoactive antimicrobial coating based on a PEDOT-fullerene C_60_ polymeric dyad. RSC Adv..

[15] Virovska D., Paneva D., Manolova N., Rashkov I., Karashanova D. (2016). Photocatalytic self-cleaning poly(L-lactide) materials based on a hybrid between nanosized zinc oxide and expanded graphite or fullerene. Mater. Sci. Eng. C.

[16] Al-Jumaili A., Alancherry S., Bazaka K., Jacob M.V. (2017). Review on the antimicrobial properties of carbon nanostructures. Materials.

[17] Díez-Pascual A.M. (2021). State of the Art in the Antibacterial and Antiviral Applications of Carbon-Based Polymeric Nanocomposites. Int. J. Mol. Sci..

[18] Moon J., Jiang H., Lee E.C. (2021). Physical Surface Modification of Carbon-Nanotube/Polydimethylsiloxane Composite Electrodes for High-Sensitivity DNA Detection. Nanomaterials.

[19] Wu S., Wang X., Li Z., Zhang S., Xing F. (2020). Recent Advances in the Fabrication and Application of Graphene Microfluidic Sensors. Micromachines.

[20] Chodkowski M., Sulym I.Y., Terpiłowski K., Sternik D. (2021). Surface Properties of Silica—MWCNTs/PDMS Composite Coatings Deposited on Plasma Activated Glass Supports. Appl. Sci..

[21] Emelyantsev S., Prazdnova E., Chistyakov V., Alperovich I. (2019). Biological Effects of C_60_ Fullerene Revealed with Bacterial Biosensor—Toxic or Rather Antioxidant?. Biosensors.

[22] Sarasamma S., Audira G., Juniardi S., Sampurna B.P., Lai Y.H., Hao E., Chen J.R., Hsiao C.D. (2018). Evaluation of the effects of carbon 60 nanoparticle exposure to adult zebrafish: A behavioral and biochemical approach to elucidate the mechanism of toxicity. Int. J. Mol. Sci..

[23] Lun-Fu A.V., Bubenchikov A.M., Bubenchikov M.A., Ovchinnikov V.A. (2021). Numerical Simulation of Interaction Between Kr+ Ion and Rotating C_60_ Fullerene Towards for Nanoarchitectonics of Fullerene Materials. Crystals.

[24] Tselukin V., Dzhumieva A., Yakovlev A., Mostovoy A., Zakirova S., Strilets A., Lopukhova M. (2021). Electrodeposition and Corrosion Properties of Nickel—Graphene Oxide Composite Coatings. Materials.

[25] Armentano I., Dottori M., Fortunati E., Mattioli S., Kenny J.M. (2010). Biodegradable polymer matrix nanocomposites for tissue engineering: A review. Polym. Degrad. Stab..

[26] Zhang N., Xu C., Azer A., Liu H. (2019). Dispersibility and characterization of polyvinyl alcohol–coated magnetic nanoparticles in poly (glycerol sebacate) for biomedical applications. J. Nanopart. Res..

[27] Jayalekshmi A.C., Victor S.P., Sharma C.P. (2013). Magnetic and degradable polymer/bioactive glass composite nanoparticles for biomedical applications. Colloids Surf. B Biointerfaces.

[28] Boval’dinova K.A., Sherstneva N.E., Fel’dshtein M.M., Moskalets A.P., Khokhlov A.R. (2019). Pressure-sensitive adhesives with tunable tackiness. Polym. Sci..

[29] Aleshina A.L., Shibaeva A.V., Philippova O.E., Khokhlova A.R. (2020). Self-healing double polymer networks with dynamic cross-links. Dokl. Akad. Nauk.

[30] Xu C., Wang Y., Wu J., Song S., Cao S., Xuan S., Gong X. (2017). Anti-impact response of Kevlar sandwich structure with silly putty core. Compos. Sci. Technol..

[31] Solomatin A.S., Tsareva Y.V., Mashchenko V.I., Savin A.V., Chigrinov V.G., Chausov D.N. (2020). New principles of organizing an interactive multi-channel visual information flow by display and projective means on the basis of ordered crystalline 4-cyano-4-octyloxybiphenyl microstructures in borosiloxane gels. Liq. Cryst. Appl..

[32] Talreja K., Chauhan I., Ghosh A., Majumdar A., Butola B.S. (2017). Functionalization of silica particles to tune the impact resistance of shear thickening fluid treated aramid fabrics. RSC Adv..

[33] Li D., Wang R., Liu X., Fang S., Sun Y. (2018). Shear-thickening fluid using oxygen-plasma-modified multi-walled carbon nanotubes to improve the quasi-static stab resistance of Kevlar fabrics. Polymers.

[34] Ermakova M.V., Mashchenko V.I., Chausova O.V., Solomatin A.S., Volosnikova N.I., Chausov D.N. (2019). Formation of ordered crystalline microstructures of 4-cyano-4-octyloxydiphenyl in borosiloxane gels. Liq. Cryst. Appl..

[35] Liu Z., Picken S.J., Besseling N.A. (2014). Polyborosiloxanes (PBSs), synthetic kinetics, and characterization. Macromolecules.

[36] Li X., Zhang D., Xiang K., Huang G. (2014). Synthesis of polyborosiloxane and its reversible physical crosslinks. RSC Adv..

[37] Palmer R.M., Green P.C. (2008). Energy Absorbing Material. U.S. Patent.

[38] Speck O., Speck T. (2019). An overview of bioinspired and biomimetic self-repairing materials. Biomimetics.

[39] Wood C.D., Green P.A. (2017). Method and Device for Detecting Fascia Damage and Repairing the Same. U.S. Patent.

[40] Tee B.C.K., Wang C., Allen R., Bao Z. (2012). An electrically and mechanically self-healing composite with pressure- and flexion-sensitive properties for electronic skin applications. Nat. Nanotechnol..

[41] Wang H., Zhu B., Jiang W., Yang Y., Leow W.R., Wang H., Chen X. (2014). A mechanically and electrically self-healing supercapacitor. Adv. Mater..

[42] Belyaev V.V., Mashchenko V.I., Chausov D.N., Solomatin A.S. (2015). A Method of Obtaining of a Mixture of Liquid Crystal with a Polymer for Display Technology and Optoelectronics. RF Patent.

[43] Mashchenko V.I., Sitnikov N.N., Khabibullina I.A., Chausov D.N., Shelyakov A.V., Spiridonov V.V. (2021). Effect of boric acid on the structure and properties of borosiloxanes. Polym. Sci..

[44] Gudkov S.V., Simakin A.V., Bunkin N.F., Shafeev G.A., Astashev M.E., Glinushkin A.P., Grinberg M.A., Vodeneev V.A. (2020). Development and application of photoconversion fluoropolymer films for greenhouses located at high or polar latitudes. J. Photochem. Photobiol..

[45] Andrievsky G.V., Klochkov V.K., Bordyuh A.B., Dovbeshko G.I. (2002). Comparative analysis of two aqueous-colloidal solutions of C_60_ fullerene with help of FTIR reflectance and UV-Vis spectroscopy. Chem. Phys. Lett..

[46] Kirsanov E.A., Timoshin Y.N. (2012). Non-Newtonian flow of structured systems. II. Analysis of flow curve. Liq. Cryst. Appl..

[47] Shcherbakov I.A., Baimler I.V., Gudkov S.V., Lyakhov G.A., Mikhailova G.N., Pustovoy V.I., Sarimov R.M., Simakin A.V., Troitsky A.V. (2020). Influence of a Constant Magnetic Field on Some Properties of Water Solutions. Dokl. Phys..

[48] Belov S.V., Lobachevsky Y.P., Danilejko Y.K., Egorov A.B., Simakin A.V., Maleki A., Temnov A.A., Dubinin M.V., Gudkov S.V. (2020). The role of mitochondria in the dual effect of low-temperature plasma on human bone marrow stem cells: From apoptosis to activation of cell proliferation. Appl. Sci..

[49] Gudkov S.V., Lyakhov G.A., Pustovoy V.I., Shcherbakov I.A. (2019). Influence of Mechanical Effects on the hydrogen peroxide concentration in aqueous solutions. Phys. Wave Phenom..

[50] Manevich Y., Held K.D., Biaglow J.E. (1997). Coumarin-3-Carboxylic Acid as a Detector for Hydroxyl Radicals Generated Chemically and by Gamma Radiation. Radiat. Res..

[51] Baimler I., Simakin A., Uvarov O., Volkov M., Gudkov S. (2020). Generation of hydroxyl radicals during laser breakdown of aqueous solutions in the presence of Fe and Cu nanoparticles of different sizes. Phys. Wave Phenom..

[52] Holley A.E., Cheeseman K.H. (1993). Measuring free radical reactions in vivo. Br. Med. Bull..

[53] Gudkov S.V., Garmash S.A., Shtarkman I.N., Chernikov A.V., Karp O.E., Bruskov V.I. (2010). Long-lived protein radicals induced by X-ray irradiation are the source of reactive oxygen species in aqueous medium. Dokl. Biochem. Biophys..

[54] Sharapov M.G., Novoselov V.I., Penkov N.V., Fesenko E.E., Vedunova M.V., Bruskov V.I., Gudkov S.V. (2019). Protective and adaptogenic role of peroxiredoxin 2 (Prx2) in neutralization of oxidative stress induced by ionizing radiation. Free Radic. Biol. Med..

[55] Bruskov V.I., Gaziev A.I., Malakhova L.V., Mantsygin I., Morenkov O.S. (1996). Monoclonal antibodies to 8-oxo-2′-deoxyguanosine (8-hydroxyguanosine). Characteristics and use for determining DNA damage by active forms of oxygen. Biochemistry.

[56] Garmash S.A., Smirnova V.S., Karp O.E., Usacheva A.M., Berezhnov A.V., Ivanov V.E., Chernikov A.V., Bruskov V.I., Gudkov S.V. (2014). Pro-oxidative, genotoxic and cytotoxic properties of uranyl ions. J. Environ. Radioact..

[57] Tuttle A.R., Trahan N.D., Son M.S. (2021). Growth and maintenance of *Escherichia coli* laboratory strains. Curr. Protoc..

[58] Barkhudarov E.M., Kossyi I.A., Anpilov A.M., Ivashkin P.I., Artem’ev K.V., Moryakov I.V., Misakyan M.A., Christofi N., Burmistrov D.E., Smirnova V.V. (2020). New nanostructured carbon coating inhibits bacterial growth, but does not influence on animal cells. Nanomaterials.

[59] Gudkov S.V., Simakin A.V., Konushkin S.V., Ivannikov A.Y., Nasakina E.O., Shatova L.A., Kolmakov A.G., Sevostyanov M.A. (2020). Preparation, structural and microstructural characterization of Ti–30Nb–10Ta–5Zr alloy for biomedical applications. J. Mater. Res. Technol..

[60] Gudkov S.V., Simakin A.V., Sevostyanov M.A., Konushkin S.V., Losertová M., Ivannikov A.Y., Kolmakov A.G., Izmailov A.Y. (2020). Manufacturing and study of mechanical properties, structure and compatibility with biological objects of plates and wire from new Ti-25Nb-13Ta-5Zr alloy. Metals.

[61] Sevostyanov M.A., Kolmakov A.G., Sergiyenko K.V., Kaplan M.A., Baikin A.S., Gudkov S.V. (2020). Mechanical, physical-chemical and biological properties of the new Ti-30Nb-13Ta-5Zr alloy. J. Mater. Sci..

[62] Borisov S.N., Voronkov M.G., Lukewitz E.Y. (1966). Organoelement Compounds. Derivatives of Inorganogens.

[63] Canaparo R., Foglietta F., Limongi T., Serpe L. (2021). Biomedical applications of reactive oxygen species generation by metal nanoparticles. Materials.

[64] Abdal Dayem A., Hossain M.K., Lee S.B., Kim K., Saha S.K., Yang G.M., Choi H.Y., Cho S.G. (2017). The role of reactive oxygen species (ROS) in the biological activities of metallic nanoparticles. Int. J. Mol. Sci..

[65] Henry T.B., Petersen E.J., Compton R.N. (2011). Aqueous fullerene aggregates (nC_60_) generate minimal reactive oxygen species and are of low toxicity in fish: A revision of previous reports. Curr. Opin. Biotechnol..

[66] Akhtar M.J., Ahamed M., Alhadlaq H.A., Alshamsan A. (2017). Mechanism of ROS scavenging and antioxidant signalling by redox metallic and fullerene nanomaterials: Potential implications in ROS associated degenerative disorders. Biochim. Biophys. Acta.

[67] Dugan L.L., Lovett E.G., Quick K.L., Lotharius J., Lin T.T., O’malley K.L. (2001). Fullerene-based antioxidants and neurodegenerative disorders. Parkinsonism Relat. Disord..

[68] Andrievsky G.V., Bruskov V.I., Tykhomyrov A.A., Gudkov S.V. (2009). Peculiarities of the antioxidant and radioprotective effects of hydrated C_60_ fullerene nanostuctures in vitro and in vivo. Free Radic. Biol. Med..

[69] Yin J.J., Fu P.P., Lutterodt H., Zhou Y.T., Antholine W.E., Wamer W. (2012). Dual role of selected antioxidants found in dietary supplements: Crossover between anti- and pro-oxidant activities in the presence of copper. J. Agric. Food. Chem..

[70] Gudkov S.V., Guryev E.L., Gapeyev A.B., Sharapov M.G., Bunkin N.F., Shkirin A.V., Zabelina T.S., Glinushkin A.P., Sevost’yanov M.A., Belosludtsev K.N. (2019). Unmodified hydrated C_60_ fullerene molecules exhibit antioxidant properties, prevent damage to DNA and proteins induced by reactive oxygen species and protect mice against injuries caused by radiation-induced oxidative stress. Nanomedicine.

[71] Matsuda S., Matsui S., Shimizu Y., Matsuda T. (2011). Genotoxicity of colloidal fullerene C_60_. Environ. Sci. Technol..

[72] Totsuka Y., Kato T., Masuda S.I., Ishino K., Matsumoto Y., Goto S., Kawanish M., Yagi T., Wakabayashi K. (2011). In vitro and in vivo genotoxicity induced by fullerene (C_60_) and kaolin. Genes Environ..

[73] Bernstein R., Prat F., Foote C.S. (1999). On the mechanism of DNA cleavage by fullerenes investigated in model systems: Electron transfer from guanosine and 8-oxo-guanosine derivatives to C_60_. J. Am. Chem. Soc..

[74] Gao J., Wang H.L., Shreve A., Iyer R. (2010). Fullerene derivatives induce premature senescence: A new toxicity paradigm or novel biomedical applications. Toxicol. Appl. Pharmacol..

[75] Takahashi M., Kato H., Doi Y., Hagiwara A., Hirata-Koizumi M., Ono A., Kubota R., Nishimura T., Hirose A. (2012). Sub-acute oral toxicity study with fullerene C_60_ in rats. J. Toxicol. Sci..

[76] Sumner S.C., Snyder R.W., Wingard C., Mortensen N.P., Holland N.A., Shannahan J.H., Dhungana S., Pathmasiri W., Han L., Lewin A.H. (2015). Distribution and biomarkers of carbon-14-labeled fullerene C_60_ ([^14^C(U)]C_60_) in female rats and mice for up to 30 days after intravenous exposure. J. Appl. Toxicol..

[77] Chen Q., Ma Z., Liu G., Wei H., Xie X. (2016). Antibacterial activity of cationic cyclen-functionalized fullerene derivatives: Membrane stress. Dig. J. Nanomater. Biostruct..

[78] Zhang Y., Zhang H., Zou Q., Xing R., Jiao T., Yan X. (2016). An injectable dipeptide-fullerene supramolecular hydrogel for photodynamic antibacterial therapy. J. Mater. Chem. B.

[79] Bai Y., Wu X., Ouyang P., Shi M., Li Q., Maimaiti T., Lan S., Yang S., Chang X. (2021). Surface modification mediates the interaction between fullerene and lysozyme: Protein structure and antibacterial activity. Environ. Sci. Nano.

[80] Aquino A., Chan J., Giolma K., Loh M. (2010). The effect of a fullerene water suspension on the growth, cell viability, and membrane integrity of *Escherichia coli* B23. Mater. Sci..

[81] Mashino T., Nishikawa D., Takahashi K., Usui N., Yamori T., Seki M., Endo T., Mochizuki M. (2003). Antibacterial and antiproliferative activity of cationic fullerene derivatives. Bioorg. Med. Chem. Lett..

[82] Li D., Lyon D.Y., Li Q., Alvarez P.J. (2008). Effect of soil sorption and aquatic natural organic matter on the antibacterial activity of a fullerene water suspension. Environ. Toxicol. Chem..

[83] Skariyachan S., Parveen A., Garka S. (2017). Nanoparticle Fullerene (C_60_) demonstrated stable binding with antibacterial potential towards probable targets of drug resistant *Salmonella typhi*—A computational perspective and in vitro investigation. J. Biomol. Struct. Dyn..

[84] Ghabdian Y., Taheri A., Jahanian-Najafabadi A. (2021). Development of novel topical formulation from fullerene with antibacterial activity against *Propionibacterium acnes*. Fuller. Nanotub. Carbon Nanostruct..

[85] Tollas S., Bereczki I., Sipos A., Rőth E., Batta G., Daróczi L., Kéki S., Ostorházi E., Rozgonyi F., Herczegh P. (2012). Nano-sized clusters of a teicoplanin ψ-aglycon-fullerene conjugate. Synthesis, antibacterial activity and aggregation studies. Eur. J. Med. Chem..

[86] Prylutska S.V., Matyshevska O.P., Golub A.A., Prylutskyy Y.I., Potebnya G.P., Ritter U., Scharff P. (2007). Study of C_60_ fullerenes and C_60_-containing composites cytotoxicity in vitro. Mater. Sci. Eng. C.

[87] Misra C., Thotakura N., Kumar R., Singh B., Sharma G., Katare O.P., Raza K. (2017). Improved cellular uptake, enhanced efficacy and promising pharmacokinetic profile of docetaxel employing glycine-tethered C_60_-fullerenes. Mater. Sci. Eng. C.

